# Current Landscape and Evolving Therapies for Primary Biliary Cholangitis

**DOI:** 10.3390/cells13181580

**Published:** 2024-09-19

**Authors:** Stefano Fiorucci, Ginevra Urbani, Cristina Di Giorgio, Michele Biagioli, Eleonora Distrutti

**Affiliations:** 1 Dipartimento di Medicina e Chirurgia, Università di Perugia, 06123 Perugia, Italy; ginevra.urbani@dottorandi.unipg.it (G.U.); cristina.digiorgio@unipg.it (C.D.G.); michele.biagioli@unipg.it (M.B.); 2 SC di Gastroenterologia ed Epatologia, Azienda Ospedaliera di Perugia, 06123 Perugia, Italy; eleonora.distrutti@ospedale.perugia.it

**Keywords:** cholangiocytes, cholestasis, farnesoid-x-receptor (FXR), macrophages, peroxisome-proliferator-associated receptors (PPAR), T cells

## Abstract

Primary Biliary Cholangitis (PBC) is a chronic autoimmune liver disorder characterized by progressive cholestatic that, if untreated, can progress to liver fibrosis, cirrhosis and liver decompensation requiring liver transplant. Although the pathogenesis of the disease is multifactorial, there is a consensus that individuals with a genetic predisposition develop the disease in the presence of specific environmental triggers. A dysbiosis of intestinal microbiota is increasingly considered among the potential pathogenic factors. Cholangiocytes, the epithelial cells lining the bile ducts, are the main target of a dysregulated immune response, and cholangiocytes senescence has been recognized as a driving mechanism, leading to impaired bile duct function, in disease progression. Bile acids are also recognized as playing an important role, both in disease development and therapy. Thus, while bile acid-based therapies, specifically ursodeoxycholic acid and obeticholic acid, have been the cornerstone of therapy in PBC, novel therapeutic approaches have been developed in recent years. In this review, we will examine published and ongoing clinical trials in PBC, including the recently approved peroxisome-proliferator-activated receptor (PPAR) agonist, elafibranor and seladelpar. These novel second-line therapies are expected to improve therapy in PBC and the development of personalized approaches.

## 1. Introduction

Primary Biliary Cholangitis (PBC), formerly Primary Biliary Cirrhosis, is a progressive cholestatic disease that if untreated might progress to cirrhosis and liver decompensation requiring liver transplant [1]. PBC is considered an organ-specific autoimmune disorder and, along with the autoimmune hepatitis (AIH) and the primary sclerosing cholangitis (PSC), is classified as an autoimmune liver disease. In contrast, however, to AIH and PSC, the treatment paradigm for PBC is based on bile acids, such as ursodeoxycholic acid and obeticholic acid (OCA), a Farnesoid-X-Receptor (FXR) agonist, and Peroxisome Proliferator-Activated Receptor (PPAR)s ligands [2], although these agents have no effects on AIH, where the cornerstone therapy is the immunosuppression, and no licensed drug have been proven effective in slowing disease progression in PSC [3,4]. Supporting the view that PBC should be considered as an autoimmune disease, there is its high gender specificity (approx. 90% of patients are female), the positivity to disease-specific anti-mitochondrial autoantibodies (AMAs), the prototypical liver histopathology and frequent concomitance with other autoimmune diseases (e.g., Hashimoto’s thyroiditis, rheumatoid arthritis) [5,6]. The incidence of PBC in the European Union (EU), the USA and Canada, Asia and Australia range between 0.9 and 5.8 per 100,000 population per year, while the prevalence is 1.9–40.2 per 100,000. In Western countries, it is estimated that 1 in 1000 women over the age of 40 live with PBC [7]. The prevalence of PBC has witnessed a constant increase in the last two decades in the EU, North America and the Asia-Pacific regions while the quality of life is improving along with a reduction in liver transplants and mortality [8].

PBC is a progressive hepatic disease and the early stages of the disease manifest with fatigue, pruritus, right upper quadrant abdominal discomfort and jaundice. The diagnosis of PBC, in these early stages, is confirmed when at least two out of the following three criteria are satisfied: (1) biochemical indicators of cholestasis, particularly elevated levels of alkaline phosphatase (ALP); (2) detection of antimitochondrial antibodies (AMA) or other PBC-specific autoantibodies, such as sp100 or sp210, if AMA is not detected (<10%); and, (3) typical histopathology features at liver biopsy [9,10,11,12].

The introduction of ursodeoxycholic acid (UDCA), a secondary bile acid normally present in human bile at low concentrations, (<3% of total bile acids) [3,4], in the treatment of PBC patients has changed the disease course, improving transplant-free survival rates to up to 60% of patients, thereby reducing mortality. Currently, life expectancy in PBC patients under UDCA therapy is similar to non-PBC patients.

## 2. Pathogenic Mechanisms in PBC

**Figure 1:**
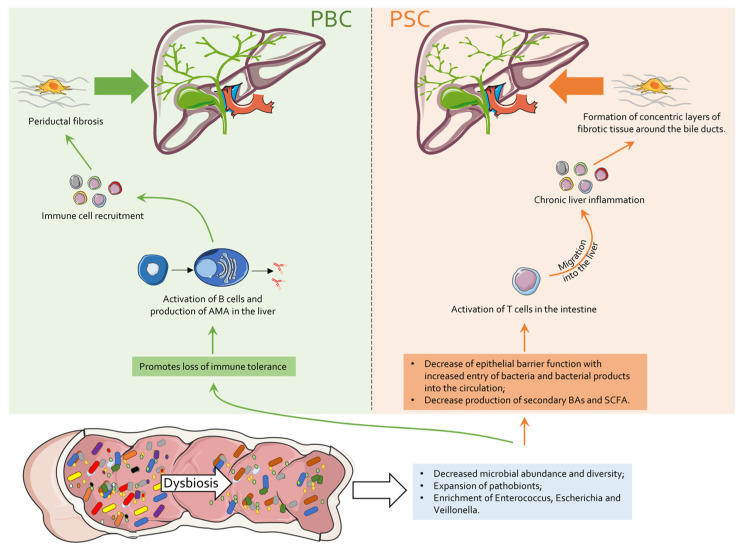
Gut-driven pathogenesis of PBC and PSC. Gut microbiota dysbiosis, characterized by a reduction in microbial abundance and diversity, as well as pathobiont increase, represents the first step in both PBC and PSC onset. Specifically, in PBC, such a condition promotes the loss of immune tolerance with subsequent activation of B cells and AMA production directed against intrahepatic cholangiocytes; further immune cell recruitment and cytokine secretion, then, contribute to periductal fibrosis. In PSC, microbiota dysbiosis entails a decreased production of secondary bile acids and short-chain fatty acids (SCFAs) as well as reduced epithelial barrier function resulting in increased entry of bacteria into the circulation; this induces intestinal T-cell activation, the initiation of chronic liver inflammation and the formation of the characteristics of an “onion-ring”, concentric layers of fibrotic tissue around bile ducts.

Although the exact etiology of PBC remains elusive, it is generally accepted that in the presence of a permissive genetic background, exposure to certain environmental factors may lead to the presentation of the E2 subunits of the pyruvate dehydrogenase complex (PDC-E2) to antigen-presenting cells. This early step is then followed by the expansion of both innate and adaptive immunity that plays a role in bile duct destruction, relapse and chronic progression. Because the PDC-E2 is expressed by cholangiocytes, the immune response is directed toward these cells resulting in injury biliary to epithelial cells, chronic cholestasis and periductular fibrosis that might progress to liver fibrosis (Figure 1).

### 2.1. Genetics of PBC

Despite precise genetic associations still not having been completely unveiled, polymorphisms in a Major Histocompatibility Complex (MHC)—Human Leukocyte Antigens (HLA) in humans—might be responsible for the augmented immunogenicity of the self-peptide-MHC complex interacting with T-cell co-receptors [13,14]. In particular, for HLA class II antigens, HLA DRw8 incidence was found to be significantly increased in PBC patients compared with controls (36% vs. 3.6%) [15]. Similarly, the presence of the DRB1*0801-DQA1*0401/0601-DQB1*04 haplotype is also increased among woman living with PBC compared with non-PBC subjects. Conversely, there is lower incidence of DRB1*1501-DQA1*0102-DQB1*0602 and DRB1*1302-DQA1*0102-DQB1*0604 haplotypes in women living with PBC, highlighting a potential protective role against disease development [16].

Single nucleotide polymorphisms (SNPs) of genes encoding for molecules involved in the immune response such as cytotoxic T-lymphocyte antigen-4 (CTLA-4) [17], interleukin-1 (IL-1) [18] and IL-10 [19] seem to be associated with PBC susceptibility. Alterations in genes involved in B-cell activation and autoantibodies production, such as POU domain class 2-associating factor 1 (POU2AF) and Spi-B transcription factor (SPIB), were found to be associated with PBC [20].

The first Genome-Wide Association Study (GWAS) carried out in the USA and Canada has detected a robust association between PBC and some genetic variants of IL-12 and IL-12 receptor β2 genes. Of relevance, these receptors drive a Th1 polarization of CD4^+^ T cells, further supporting a role for auto-immunity [21]. Nevertheless, the same association was not found in another study performed on an Asian cohort, which identified the tumor necrosis factor (TNF) superfamily member 15 (TNFSF15) as a potential risk locus for PBC development [22]. These discrepancies among different ethnic groups sharing the same genetic background point to the important influence of environmental factors in PBC pathogenesis.

### 2.2. Epigenetics and Environmental Factors

Despite genetics having a critical impact on disease onset and development, there is a consensus that individuals with genetic predisposition develop the disease in the presence of specific environmental triggers [23].

Multiple epidemiological studies correlated *E. coli*-derived recurrent urinary tract infections (rUTI) to PBC onset, postulating the microorganism (the most prevalent isolated in women with rUTI) to be a possible trigger for disease development [24]: microbial infection seems to have a role in AMA induction through a mechanism of molecular mimicry, i.e., the presence of cross-reactivity between infectious and self-epitopes [25]. In particular, human PDC-E2, a non-covalent enzymatic complex located on the inner mitochondrial membrane that catalyzes the oxidative decarboxylation of pyruvate to acetyl CoA [26], is molecularly similar to *E. coli* PDC-E2, thus being responsible for immunological cross-reactivity and the breakdown of tolerance to mitochondrial autoantigens [27].

Another microorganism deemed to be involved in PBC development through molecular mimicry and cross-reactivity towards PDC-E2 is *Novosphingobium aromaticivorans*, a ubiquitous gram-negative xenobiotic-metabolizing bacterium containing lipoylated proteins 100- to 1000-fold more reactive than those of *E. coli* [28]. A study from Selmi et al. reported that in sera from 100% of anti-PDC-E2 (77/77) positive PBC patients were found antibodies against *Novosphingobium aromaticivorans*, providing further support for the concept that exposure to *Novosphingobium aromaticivorans* could be a trigger for PBC development [28,29].

Extensive epidemiological data suggest that xenobiotics are likely to be involved in PBC development: a study by Trivedi et al. showed how 2-octynamide, derived from 2-octynoic acid (a cosmetics ingredient), mimics the structure of the PDC-E2 immunodominant autoantigen [11]. Moreover, frequent use of nail polish and a history of smoking or hormone replacement therapy were also found to be significantly correlated to PBC onset while, on the contrary, never having been pregnant was significantly associated with protection from disease development [30].

## 3. The Immunology of PBC

**Figure 2:**
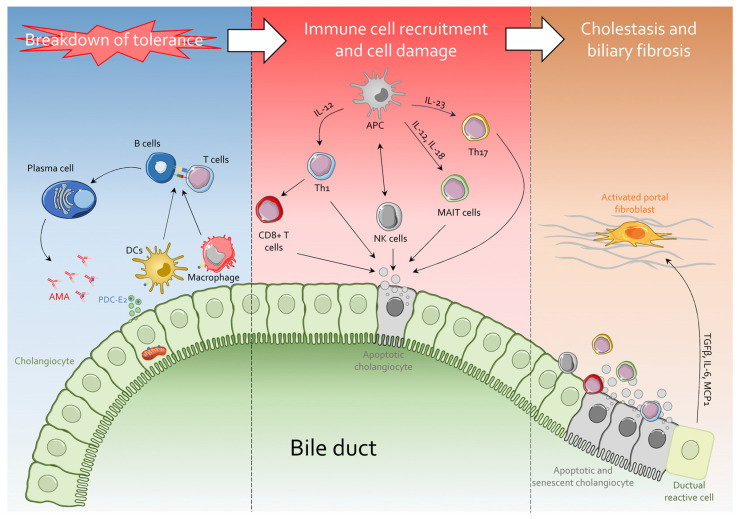
Breakdown of tolerance has a pivotal role in PBC pathogenesis. Aberrant expression of PDC-E2 subunit by cholangiocytes causes the recruitment of B cells, T cells and DCs promoting anti-PDC-E2 autoantibody secretion. Pro-inflammatory cytokine secretion and further immune cell recruitment (e.g., CD8^+^ T and NK cells) induce cholangiocytes senescence and apoptosis, responsible for biliary cholestasis. Cholangiocytes’ senescence-associated secretory phenotype (SASP) onset, finally, promotes portal fibroblast activation and subsequent biliary fibrosis. MAIT, mucosal-associated invariant T (MAIT) cells.

As mentioned above, PBC is characterized by high titers of AMAs and progressive intrahepatic cholestasis due to inflammatory cholangitis (Figure 2).

Pathognomonic anti-PDC-E2 autoantibodies, present in at least 95% of PBC patients [31], result from autoreactive B-cell and T-cell responses directed against mitochondrial autoantigens. Interestingly, recent epitope mapping studies revealed that autoreactivity is specifically directed to the C-terminus of the molecule, where the enzymatic catalytic active site is located [32]. Secretory IgAs anti-PDC-E2 have been found in the saliva, bile and urine of PBC patients [33]. However, it is still not clear why, although the antigen is expressed in all nucleated cells, the autoimmune response is restricted to the intra-hepatic bile ducts. Although present in only about 30% of PBC patients, anti-nuclear antibodies (ANAs) represent another characteristic feature of the disease. ANA are more frequently detected in the AMA-negative PBC patients [34]. The anti-nuclear pore complex (NPCs) autoantibodies such as *gp210* and *p62* were also detected in AMA-negative woman living with PBC and associates to a more aggressive disease [35].

Typical PBC organ-specific damage limited to the liver and characterized by chronic progressive destruction of small intrahepatic bile ducts with cholestasis, portal inflammation and, ultimately, fibrosis is mainly caused by the liver accumulation (Figure 1 and Figure 2) of autoreactive CD4^+^ and CD8^+^ T cells present both in peripheral blood and hepatic infiltrating cells [36]. Again, as for humoral response, T-cell responses appear to be principally directed against the PDC-E2 subunit [37]: recent data from murine models of PBC have suggested that a critical mechanism of biliary destruction is actually mediated by liver-infiltrating CD8^+^ T cells [38]. T_H_1 and T_H_17 cells accumulate around damaged bile ducts of inflamed portal tracts in PBC patients, leading to the release of pro-inflammatory cytokines and chemokines and growth factors (Figure 2), reflecting the formation of a pro-inflammatory microenvironment [39,40]. In contrast, low levels of circulating CD4^+^ CD25^high^ regulatory T cells (T_reg_), which mediate the immune system self-tolerance and are essential for autoimmune disease prevention, are detected in patients with PBC compared with controls [41]. In addition, mucosal-associated invariant T (MAIT) cells also play a role in the in the pathogenesis of PBC. MAIT cells accumulate in the liver through CXCL12-CXCR4-mediated chemotaxis, leading to the production of pro-inflammatory cytokines and contributing to portal inflammation, potentially driven by increased levels of IL-18.

## 4. Immunobiology of Cholangiocytes in PBC

Cholangiocytes, or biliary epithelial cells (BECs), are postmitotic ciliated cuboidal epithelial cells that form the lining of the intra- and extrahepatic biliary ducts [42]. Although being a relatively rare hepatic cell type (~4% of the total liver parenchymal cells), BECs play a pivotal role in primary bile production and modification via absorption and the secretion of ions, solutes and water as it is transported along the biliary tree [43,44]. Moreover, several studies confirmed BECs’ ability to represent the first line of defense against luminal microbes in the biliary system thanks to the expression of a variety of pathogen-recognition receptors (PRRs) such as toll-like receptors (TLRs) and nucleotide-binding oligomerization domain proteins (NODs) [45,46]. Recognizing and discriminating both structurally conserved pathogen-associated molecular patterns (PAMPs) and danger-associated molecular patterns (DAMPs) released by damaged liver cells, BECs work in all respects as mediators of the innate immune response [47]. In response to infectious, toxic, inflammatory or autoimmune challenges, cholangiocytes transition from a quiescent state to an activated state, known as ‘reactive cholangiocytes’ (Figure 2). Upon the activation of the NF-κB pathway, these cells begin to proliferate and secrete various proinflammatory and profibrogenic factors described in Figure 2. These are mediators in both paracrine and autocrine manners [48,49,50,51]. IL-6 facilitates the proliferation of BECs and promotes B-cell differentiation and immunoglobulin secretion [52,53]. TNF-α enhances the expression of adhesion molecules ((Figure 2) on BECs and augments the functions of cytotoxic T lymphocytes, and drives an apoptotic damage to the bile ducts [54,55]. The antimicrobial activity of BECs is further mediated through the secretion of human β-defensin 1 (HBD-1) and HBD-2 [56,57,58].

Such a condition persists unless the initial insult is removed: otherwise, ongoing stimulation caused by a persistent liver injury provokes progression towards fibrosis, cholestasis and, at last, malignant transformation [50]. Cholangiocyte-released TGF-β promotes the myofibroblastic differentiation of portal fibroblasts and hepatic stellate cells (HSCs) [59] and regulates extracellular matrix (ECM) deposition by nearby mesenchymal cells [60]; similarly, MCP-1 induces myofibroblast differentiation and collagen-1 release by portal fibroblasts as a result of chronic inflammation [49]. Finally, a loss of tolerance to the mitochondrial antigen PDC-E2 represents the trigger event for the immune-pathogenesis of PBC. In particular, it seems that pro-inflammatory cytokines could enhance the expression of microRNA-506 (miR-506) in BECs, resulting in the overexpression and mislocalization of PDC-E2 in PBC patients [61] followed by NKT cell, MAIT cell, and CD4^+^ and CD8^+^ cell infiltration around intrahepatic bile ducts [62].

Cholestasis is a major clinical feature of PBC. Due to altered cholangiocytes’ physiology and hyperproliferation, leading to bile ducts obstruction [63], bile cannot be properly synthesized nor excreted into the duodenum and accumulates inside hepatocytes, leading to high bilirubin blood levels, pruritus, osteodystrophy and fatigue, in addition to contributing to liver cell injury with the accumulation of hydrophobic and potentially toxic bile acids [64]. PBC patients develop a profound alteration in their bile acid structure when compared with healthy controls, characterized by a decreased conversion of primary to secondary bile acids, indicating the impaired microbial metabolism of intestinal bile acids by the intestinal microbiota. In particular, it has been recognized that deoxycholic acid (DCA) levels are inversely correlated with PBC-enriched gut microbes (e.g., *Veillonella*, *Klebsiella*), while being positively correlated with control-enriched microbes (e.g., *Faecalibacterium*, *Oscillospira*) [65]. Since DCA is an endogenous ligand for the G protein-coupled bile acid receptor (GPBAR1, also known as TGR5), this alteration might have a mechanistic relevance in the development of immune dysregulation in PBC [66,67].

### Apoptosis and Senescence of Cholangiocytes

In most cases, however, biliary proliferation halts and senescence or apoptotic mechanisms become prevalent with the development of ductopenia [68], a condition typical of cholangiopathies (i.e., a category of chronic liver diseases sharing cholangiocytes as a central target) such as PBC [69]. Both mechanisms, which are mutually exclusive, contribute to disease development [70].

Apoptosis is a pathway of programmed cell death occurring regularly in order to maintain the homeostatic balance between cell formation and cell death rates [71]. Actually, two apoptotic pathways can be identified: the intrinsic, activated by intracellular triggers like mitochondrial stress or unfolded protein response (UPR), and the extrinsic one, initiated by the interaction between ‘death receptors’ and their ligands (TNFα, FasL and a tumor necrosis factor-related apoptosis-inducing ligand, TRAIL) [72]. Despite the two pathways differing from each other for the initial triggering events, they converge to a common final pathway regulated by a series of cleavage-activated caspases [73]. Differently from necrosis, characterized by the spillage of intracellular content into the surrounding tissue with subsequent inflammation and damage, apoptosis produces membrane-bound vesicles derived from the disassembly of apoptotic cells and known as apoptotic bodies that are then removed by macrophages through phagocytosis [74,75]. Multiple studies have demonstrated a hyperactivation of apoptosis in BECs of PBC patients if compared with healthy controls, detecting higher expressions of TNFα, Fas, FasL and TRAIL [76,77,78]. Moreover, the overexpression of caspase-3 and -8 as well as that of proinflammatory CXCL9 and CXCL10 in the liver tissue of PBC patients has been demonstrated too [79,80], while in vitro studies have shown UDCA’s ability to inhibit apoptosis via the induction of several pro-survival pathways [81]. Bile acid pool alteration, typical of PBC patients, together with a reduction in bicarbonate umbrella secretion by the bile duct cells, cause BECs to be more sensitive to apoptosis induced by cytotoxic hydrophobic BAs [7]. As mentioned before, PBC patients are typically characterized by gut microbiota dysbiosis, with high levels of *Firmicutes* and *Proteobacteria* and reduced levels of *Bacteroidetes* [82]: this has been associated with increased BEC apoptosis through TLR2 signaling activation [83]. Moreover, the levels of the tumor suppressor protein p53 (referred to as “the guardian of the genome”) and p53-induced apoptosis are enhanced in PBC patients [84].

On the other side, cellular senescence is a phenomenon in which proliferating cells enter a cell cycle arrest, being permanently blocked in the G1 or G2 phase, unable to grow but still metabolically active [70]. Senescent cells are resistant to apoptosis thanks to the over-expression of anti-apoptotic mediators (e.g., Bcl-2, Bcl-xL) or the reduced expression of pro-apoptotic ones, as happens in PBC BECs [85]. Senescence is physiologically triggered by DNA damage, with telomere shortening occurring after repetitive cell divisions (ageing) or other stress signals: however, particular conditions can push cells into premature senescence, playing a fundamental role in the progression of some diseases such as diabetes, cardiac and end-stage liver diseases, cholestasis included [86]. Senescence cells have deleterious effects on the surrounding microenvironment via the acquisition of a senescence-associated secretory phenotype (SASP), or Senescence-Messaging Secretome (SMS) [87]. In particular, cells undergo profound changes in protein expression and secretion, starting to produce soluble factors (interleukins, chemokines and grow factors), proteases and ECM components able to modify adjacent tissues structure as well as to recruit inflammatory mediators [88]. Endoplasmic reticulum (ER) stress markers, glucose-regulated protein 78 (GRP78) and protein disulfide isomerases (PDI), were significantly increased in in vitro models of PBC and correlated with premature BEC senescence: both conditions were significantly suppressed following a pretreatment with UDCA [89]. Sasaki et al. demonstrated that BECs derived from damaged bile ducts of PBC patients are characterized by SASP, an increased expression of senescence-associated β-galactosidase (SA-βGal), a significant reduction in telomere length and multiple gammaH2AX-DNA-damage-foci, features absent both in the BECs of normal livers and chronic viral hepatitis [90]. Increased levels of cyclin-dependent kinase inhibitors p16 and p21 (promoting cell cycle arrest in G1 phase) are detected in senescent BECs of bile ducts derived by PBC patients when compared with healthy controls: moreover, the expression of p16 and p21 was much more enhanced in later (3-4) than in earlier (1-2) PBC stages, confirming the pivotal role of cholangiocytes senescence in disease progression [91].

## 5. Bile Acid-Regulated Receptors in PBC: From Pathogenesis to Therapy

As mentioned above, the cornerstone of PBC therapy is bile acid-derived agents. The introduction of ursodeoxycholic acid (UDCA), a secondary bile acid (BA) normally present in human bile (low concentration, 3% of total BAs) [3,4], in PBC treatment has changed the disease course, improving transplant-free survival rates to up to 60% of patients. Multiple studies, indeed, confirmed UDCA to be an important agonist for GPBAR1, the receptor responsive to primary (cholic acid, CA, and chenodeoxycholic acid, CDCA, synthesized by hepatocytes) and secondary (deoxycholic acid, DCA, and lithocholic acid, LCA, derivatives of primary BA metabolism operated by intestinal microbiota) bile acids and expressed by BECs lining small and large intrahepatic ducts, extrahepatic ducts and gallbladder epithelium [92,93]. In the liver, GPBAR1 activation is fundamental for hepatic homeostasis and results in (i) the increased cystic fibrosis transmembrane conductance regulator (CFTR)-dependent chloride and bicarbonate secretion, enhancing choleresis and protecting hepatic parenchyma from BA toxicity through the so called “bicarbonate umbrella” [94]; (ii) the increased expression and phosphorylation of junctional adhesion molecule A (JAM-A), thus regulating and stabilizing biliary epithelial barrier function—both in vivo and in vitro—as well as protecting against bile leakage [95]; (iii) the secretion of vasodilatory molecules (e.g., nitric oxide, NO) and the inhibition of vasoconstrictor ones (e.g., endothelin-1, ET-1) by sinusoids, contributing to liver microcirculation modulation and portal hypertension mitigation [96,97]. Moreover, the activation of GPBAR1 on Kupffer cells decreases the NF-κB-dependent inflammatory response, thus reducing hepatic inflammation [98,99,100]. At a systemic level, GPBAR1 is expressed by circulating tissue resident myeloid cells and dendritic cells (DCs), and the activation promotes a macrophage polarization towards an anti-inflammatory phenotype (M2) as well as the inhibition of maturation and the differentiation of DCs and Natural Killer T (NKT cells) [101,102]. GPBAR1 is therefore essential for the development of immune tolerance [103].

UDCA at a dose of 13–15 mg/kg/die is the first-line therapy for PBC [104]. The mechanism of cation of UDCA remains elusive, but over the years, several potential beneficial effects have been reported, including choleretic activity, the promotion of bicarbonate secretion and the displacement of endogenous hydrophobic hepatotoxic BAs by expanding the hydrophilic pool modulating BA homeostasis, maintaining the integrity of the biliary tree epithelial barrier, inhibiting the pro-inflammatory NF-κB pathway, helping in the management of PBC symptoms (i.e., pruritus, jaundice, fatigue), improving liver biochemistry (normalization of total bilirubin, alkaline phosphatase and alanine transaminase) and prolonging liver transplant (LT)-free survival [100,105,106,107].

Other bile acid-based therapies include ligands for the nuclear receptors Farnesoid-X-Receptor (FXR), Vitamin D receptor (VDR) and Pregnane-X-Receptor (PXR).

BECs represent one of the three non-parenchymal cell types in the liver that express GPBAR1, alongside Kupffer cells and liver sinusoidal endothelial cells. The activation of GPBAR1 in cholangiocytes facilitates several protective mechanisms, including (a) the upregulation of cystic fibrosis transmembrane conductance regulator (CFTR)-mediated chloride and bicarbonate secretion, which strengthens the “bicarbonate umbrella” effect [94]; (b) the enhanced expression and phosphorylation of junctional adhesion molecule A (JAM-A), leading to the stabilization and regulation of the biliary epithelial barrier both in vitro and *in vivo*, thereby preventing bile leakage [95]; (c) the secretion of vasodilatory agents such as nitric oxide (NO), along with the inhibition of vasoconstrictors like endothelin-1 (ET-1) by liver sinusoidal endothelial cells, which contributes to the maintenance of hepatic microcirculation [96,97]. Moreover, the activation of GPBAR1 in Kupffer cells, which are the resident macrophages of the liver, results in reduced inflammation through the negative modulation of NF-κB [98,99,100,108]. GPBAR1 is also expressed in blood-derived macrophages and dendritic cells (DCs), where its activation promotes macrophage polarization towards an anti-inflammatory M2 phenotype and inhibits the maturation and differentiation of DCs and Natural Killer T (NKT) cells [101,102].

UDCA has been the first drug approved by the FDA for the treatment of PBC and remains the first-line therapy [104]. Clinically, UDCA at a dose of 15 mg/kg/day or higher alleviates symptoms of PBC, such as pruritus, jaundice and fatigue, improves liver biochemical markers (e.g., normalization of total bilirubin, alkaline phosphatase and alanine transaminase) and extends transplant-free survival [100,105,106,107]. Evidence suggests that UDCA may modulate GPBAR1; our in vitro studies have demonstrated that UDCA can directly transactivate GPBAR1 [92,93]. Consistent with these findings, the in vivo administration of UDCA induces GPBAR1-like effects, including enhanced bicarbonate secretion and anti-NF-κB activity, contributing to the integrity of the biliary epithelial barrier. However, other studies propose that UDCA might function as a FXR antagonist [109]. Since the therapeutic efficacy of OCA, an FXR agonist, in PBC patients is additive to UDCA, it seems unlikely that UDCA is an effective FXR antagonist in this context [93,107]. Currently, there are no clinical trials evaluating selective GPBAR1 agonists in PBC patients.

FXR is a receptor for primary bile acids [110] and is predominantly expressed in liver parenchymal cells, cholangiocytes and hepatic stellate cells (HSCs) [111]. FXR activation mitigates innate immune responses in the liver through various mechanisms: (a) FXR agonism negatively regulates NF-κB in liver-resident macrophages [112] or blood-derived leukocytes, thereby reducing the secretion of pro-inflammatory cytokines [113,114,115] and in HSCs through a pathway involving the small heterodimer partner (SHP), an FXR-regulated transcription factor [116,117,118]; (b) FXR also acts as a negative regulator of NLRP3 inflammasome activation in cholangiocytes [119]. The NLRP3 inflammasome is a multiprotein complex that detects cellular stress, triggering caspase-1 activation and the release of pro-inflammatory cytokines IL-1β and IL-18. Several studies have confirmed that FXR agonism might reverse cholestasis, and several FXR agonists have been advanced through clinical trials [120,121]. However, animal studies have shown that FXR-deficient mice are protected from cholestasis [122] and there is evidence that FXR activation might inhibit the expression activity of Multidrug Resistance Protein 4 (MRP4) [123], raising some concerns over the potential utility of FXR agonism in cholestasis, while these animal studies might support the development of anti-FXR therapies in cholestasis [124,125].

In contrast to this view, various FXR agonists have been developed to treat cholestasis. The first class of these agents, the obeticholic acid (OCA), was originally developed at the University of Perugia, in Fiorucci’s lab in 2002 [126] and was approved in 2016 by Food and Drug Administration (FDA), USA, as a second-line treatment for PBC patients who have incomplete responses to UDCA or who are intolerant to UDCA [127]. The labelled indication excludes patients with decompensated cirrhosis [127]. The efficacy of OCA in treating patients with PBC has been corroborated by numerous studies, including real-world effectiveness data from international cohorts [128]. However, ongoing clinical trials continue to assess its safety profile, particularly due to reported adverse effects in some patients, such as exacerbation of pruritus and incidents of liver decompensation or failure in cirrhotic PBC patients [111,128,129,130,131,132]. OCA has also been shown to have beneficial additive effects when used in combination with fibrates [133].

The VDR is expressed in cholangiocytes, and its activation by lithocholic acid (LCA) plays a role in modulating the innate immune response. VDR activation suppresses the proliferation and differentiation of B cells, inhibits the formation of Th17 cells and promotes the differentiation of regulatory T cells (Tregs) [134,135,136,137,138,139,140,141,142]. Studies have shown that VDR expression is reduced in PBC patients compared with healthy controls, which may contribute to disease progression [143]. Given that 3-oxo-DCA and iso-allo-LCA, in addition to LCA, may also activate VDR, further investigation of this pathway in PBC patients could be of significant importance [144,145,146].

PXR, mainly expressed by enterocytes and hepatocytes, is a well-known nuclear receptor involved in xenobiotics’ catabolism, transport and clearance via CYP3A induction [147]. PXR is expressed on a variety of tissues including bone and intestine [135] but also in immune cells [135] and cholangiocytes [136]. Rifampicin and rifaximin act as a PXR agonist, and have been used to treat cholestasis [148,149,150]. PXR acts as a receptor for LCA [138] and modulates both the innate and adaptive immune system by blocking B-cell proliferation and differentiation [138], preventing Th17 cell formation [139], while facilitating T_reg_ differentiation [140] and inhibiting the monocyte secretion of typical proinflammatory cytokines such as IL-1, IL-6, IL-8 and TNF-α [141]. Experimental cholestasis is exacerbated by PXR gene ablation [151,152] and rifampicin, a human PXR agonist, is clinically used to promote bilirubin excretion and as an itching treatment in PBC [153]. PXR also suppresses NF-κB induction in mouse models of liver injury [149,154,155].

## 6. Intestinal Microbiota in PBC

Intestinal dysbiosis is frequently observed in patients with PBC and is increasingly recognized as a contributing factor to both the onset and progression of the disease [66,156]. Using the inverse variance weighted (IVW) method, it has been demonstrated that the relative abundance of *Selenomonadales*, *Bifidobacteriales* and the genus *Lachnospiraceae*_UCG_004 is positively associated with an increased risk of developing PBC, while a higher abundance of *Peptostreptococcaceae* and *Ruminococcaceae* appears to be protective [157]. Additional studies have identified a reduction in the relative abundance of bacteria capable of producing SCFAs, such as *Faecalibacterium* spp. and *Oscillospira* spp. [158]. These microbial changes were mitigated by treatment with UDCA, indicating that UDCA may play a role in modulating the intestinal microbiota. Microbiota-derived SCFAs, particularly butyrate, may be implicated in the regulation of myeloid-derived suppressor cells (MDSCs), a subset of myeloid cells involved in PBC pathogenesis [159]. Unlike classic monocytes, MDSCs possess strong immunosuppressive abilities and inhibit the proliferation of T cells, B cells, and Natural Killer (NK) cells. Therefore, a decrease in butyrate-producing bacteria could result in impaired MDSC function and a suboptimal response to UDCA treatment, suggesting that microbiota-targeted therapies could have therapeutic potential in PBC patients.

## 7. Current Therapeutic Landscape in PBC

### 7.1. UDCA

**Figure 3:**
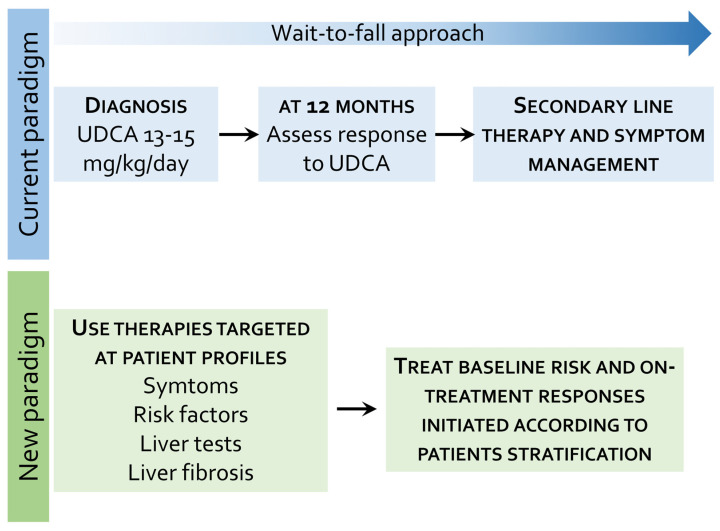
Comparison between current and new paradigms of PBC treatment. Current approaches include initiation of UDCA with assessment after 1 year of therapy and eventual shift to a second-line therapy in case of insufficient response to UDCA chosen on the basis of existing symptoms, comorbidities and drug availability. In contrast, the new paradigm is based on a more personalized approach that takes into account individual risk assessment since the beginning of the therapy.

UDCA is the standard treatment for PBC, regardless of stage of disease [160], representing the first-line treatment at a recommended dose of 13–15 mg/kg/day. The use of UDCA is associated with improved survival without liver transplantation, even among patients with an incomplete biochemical response (Figure 3).

Various scores have been developed to define responses to therapy with UDCA in PBC patients (Table 1). Biochemical analyses carried out after 6 or 12 months of therapy with UDCA have shown to be effective in providing prognostic information and predicting ongoing risk of disease progression during treatment. The biochemical treatment response is currently assessed after 12 months of therapy, focusing on ALP and bilirubin values (Figure 3).

An important consideration when starting therapy in PBC patients is also related to the severity of liver fibrosis at the start of therapy [168]. Several studies have shown that the severity of liver fibrosis is an independent risk factor for progression independent of treatment response [169]. Patients with advanced fibrosis/cirrhosis have a reduced transplant-free survival time compared with patients in the early stage of disease. Ultrasound-based noninvasive technologies such as transient elastography are of increasing importance for liver stiffness measurement (LSM) at the start of therapy and also to monitor responses to treatment in clinical trials. A baseline LSM > 15 kPa predicts a worse prognosis, whereas patients with LSM < 8 kPa have a lower risk of developing severe fibrosis under treatment [169].

### 7.2. Second-Line Therapies and Novel Approaches in PBC

**Figure 4:**
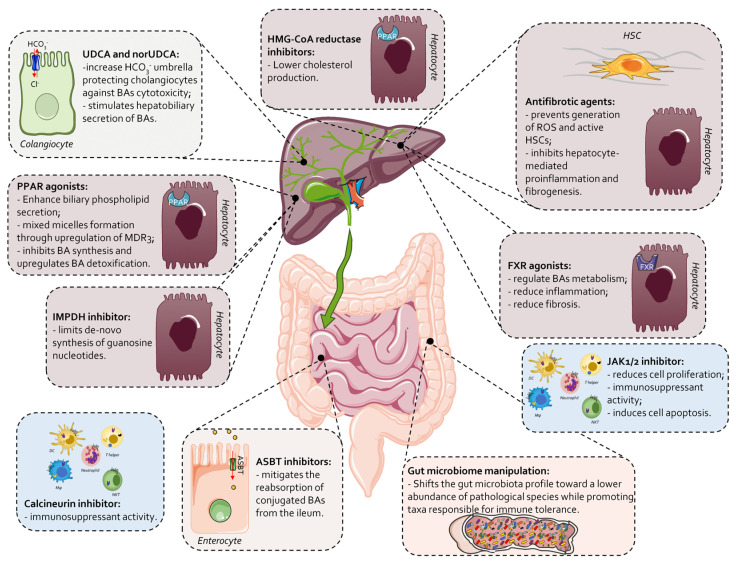
Overview of additional therapies available for the treatment of PBC patients. The therapeutic targets are multiple, corresponding to the multiple symptoms of PBC. Specifically, therapies targeting hepatocytes include the use of PPAR or FXR agonists, HMG-CoA reductase or IMPDH inhibitors and antifibrotic agents that prevent ROS generation and HSC activation. Another therapeutic target is cholangiocytes, where the use of UDCA and norUDCA increases HCO_3_^−^ production and stimulates hepatobiliary secretion of bile acids. Potential intestinal therapies involve manipulating the microbiota or using apical sodium-dependent bile acid transporter (ASBT) inhibitors to reduce bile acid reabsorption. Finally, JAK1/2 inhibitors and calcineurin inhibitors can be used to modulate the immune response.

While UDCA is currently recommended as the first-line therapy in PBC, with reassessment of efficacy after 1 year of therapy (Figure 3), a number of additional therapies are currently available or under development and more personalized approaches are likely to be developed in the near future, not only in cases of insufficient response to UDCA but also with the aim to improve quality of life and reach normalization of liver biochemistry (Table 2 and Figure 4).

These novel pharmacological approaches are directed toward a variety of consolidated or innovative molecular targets, including novel anti-itching agents [170]. Among the various treatments mentioned in Table 3, two PPAR agonists, elafibranor and seladelpar, have completed phase 3 trials and gained approval for the treatment of PBC patients in 2024 [171]. PPARs are members of the nuclear receptor family of ligand-activated transcription factors. The PPARs family include α, β, δ, and γ subtypes that are widely distributed in endocrine and non-endocrine tissues in humans. PPAR-α is mainly expressed in hepatocytes, whereas PPAR-δ has ubiquitous expression, including liver parenchymal cells, liver macrophages, HSC and BEC [172]. PPAR-α and PPAR-δ agonists have anti-inflammatory properties and affect both innate and adaptive immunity by counter-regulating the polarization of macrophages and T cells toward non-inflammatory and regulatory phenotypes (i.e., M2 and Treg) [66,173]. PPAR-α agonism induces the differentiation of regulatory T cells, whereas PPAR-δ agonism suppresses the polarization of type 17 helper T cells. Elafibranor [2,174] is a first-in-class of pan-PPAR agonists. Elafibranor activates PPAR-α, PPAR-γ and PPAR-δ in vitro, and has been developed for the treatment of PBC [175] and metabolic-associated steatotic liver disease (MASLD) [176]. On 10 June 2024, “elafibranor has received an accelerated approval based on reduction of alkaline phosphatase (ALP) in the USA for the treatment of PBC in combination with UDCA in adults who have an inadequate response to UDCA, or as monotherapy in patients unable to tolerate UDCA” (https://www.ipsen.com/press-releases/ipsens-iqirvo-receives-u-s-fda-accelerated-approval-as-a-first-in-class-ppar-treatment-for-primary-biliary-cholangitis/#:~:text=PARIS%2C%20FRANCE%2C%2010%20June%202024,acid%20(UDCA)%20in%20adults%20who accessed on 29 August 2024).

Elafibranor has also received a positive opinion over its dossier from the EMA [171]. Following elafibranor, on 14 August 2024, the FDA also granted an accelerated approval for seladelpar [177] for the treatment of PBC patients in combination with UDCA in adults who have an inadequate response to UDCA, or as monotherapy in patients unable to tolerate UDCA. The use of seladelpar is not recommended for people who have or develop decompensated cirrhosis (https://www.gilead.com/news-and-press/press-room/press-releases/2024/8/gileads-livdelzi-seladelpar-granted-accelerated-approval-for-primary-biliary-cholangitis-by-us-fda accessed on 29 August 2024).

Thus, similarly to OCA, elafibranor and seladelpar are second-line therapies in patients that do not respond or have intolerance to UDCA [178]. However, since PPARs might impact several metabolic pathways, the recent approval of these agents raises the need for an accurate stratification of patients to identify subsets that might benefit from early therapies in addition to or as a substitution for UDCA.

**Table 3:** Recently published trial in PBC.

Treatment	Clinical Trial	Treatments	End Points	Results	Ref.
**Elafibrinor** PPARα/δ	Phase 3	161 PBC adults, who had incomplete response to UDCA. Treatments: Group 1: elafibrinor 80 mg; Group 2: placebo.	Reduction in ALP levels (ALP ≥ 1.67-fold the upper limit of normal (ULN) at 52 weeks.	A biochemical response (the primary end point) was observed in 51% of the patients (55 of 108) who received elafibranor and in 4% (2 of 53) who received placebo, for a difference of 47 percentage points (95% confidence interval [CI], 32 to 57; *p* < 0.001).	[2]
**Obeticolic acid** **(OCA)**	Phase 2	Patients with PBC from POISE cohort and external control patients from Global PBC cohort and UK-PBC cohort. Treatments: Group 1: OCA (POISE cohort n = 209); Group 2: non-OCA-treated external control (Global PBC cohort n = 1381 and UK-PBC cohort n = 2135).	Evaluate time to first occurrence of liver transplantation or death in patients with OCA vs. comparable non-OCA-treated external controls.	During the 6-year follow-up, there were 5 deaths or liver transplantations in Group 1 (2.4%), 135 in the Global PBC cohort control (10.0%) and 281 in the UK-PBC control (13.2%).	[128]
	Phase 2	59 PBC patients, intolerant to UDCA. Treatments: Group 1: placebo (n = 23) Group 2: OCA 10 mg (n = 20). Group 3: OCA 50 mg (n = 16)	The percent change in ALP from baseline to the end of the double-blind phase of the study.	ALP levels were reduced in both OCA groups, respectively, by −53.9% in 10 mg group and by −37.2% in 50 mg group compared with placebo −0.8% (*p* < 0.05). Similar reductions were observed through 6 years of open-label extension treatment. **Side effects:** pruritus increased dose-dependently with OCA treatment. 15% (OCA 10 mg) and 38% (OCA 50 mg) discontinued due to pruritus.	[129]
**Linerixibat** IBAT inhibitor	Phase 2b	147 adult PBC patients with moderate to severe pruritus, numerical rating scale (NRS) ≥ 3 after 4 week of placebo treatment. Treatments: Group 1: placebo (n = 36); Group 2: linerixibat at 20 mg/d (n = 16); 90 mg/d (n = 23) 180 mg/d (n = 27); 40 mg/b.d. (n = 23); 90 mg/b.d. (n = 22), for 12 weeks (from week 4 to week 16), followed by single-blind placebo (to week 20).	Investigate dose-related changes in Mean Worst Daily Itch (MWDI) score at week 16.	At week 16, MWDI analysis showed significant differences between placebo and and linerixibat 180 mg/d (*p* < 0.05), 40 mg/b.d. (*p* < 0.05) and 90 mg/b.d. (*p* < 0.05). Diarrhea was the most frequent adverse event, and incidence increased with dose.	[179]
**Seladelpar** PPARδ	Phase 3	193 PBC patients with an inadequate response or intolerance to UDCA were enrolled. Treatments: Group 1: sedalpar 10 mg/d (n = 89); Group 3: placebo for 12 months	**Primary** composite biochemical response (ALP < 1.67 × ULN and total bilirubin ≤ ULN) at month 12.	**Primary** 61.7 % improvement in Group 1 and 41.7% in the placebo *p* < 0.05.	[177]
**Saroglitazar** PPARα/γ	Phase 2	Phase 2	37 PBC patients with UDCA resistance or intolerance. Treatments: Group 1: saroglitazar 4 mg/d (n = 13); Group 2: saroglitazar 2 mg/d (n = 14): Group 3: placebo (n = 10).	At week 16, patients from Group 1 showed a reduction of ALP levels by −163.3 U/L and Group 2 by −155.8 U/L compared to placebo (−21.1 U/L) (*p* < 0.05). Study drug was discontinued in 4 patients (3 patients in Group 1 and 1 patient in the Group 2) due to ALP increases.	[180]
**Fenofibrate** PPAR	Phase 3	117 PBC treatment-naive patients. Treatments: Group 1: UDCA; Group 2: UDCA plus fenofibrate 200 mg/d	Biochemical response percentage, according to the Barcelona criterion at 12 months.	In Group 2, 81.4% of patients achieved the primary outcome and 64.3% in Group 1 achieved the primary outcome (*p* < 0.05). There was no difference between the 2 groups in liver fibrosis and biochemical markers.	[181]
**Rituximab** Anti-CD20	Phase 3	57 aged 18-years-old or older patients with PBC and moderate to severe fatigue. Treatments: Group 1: rituximab 1000 mg/b.d.; Group 2: placebo	**Primary** Measurement of fatigue severity using the PBC-40 fatigue domain at 3 months.	**Primary** Improvement in fatigue score was seen in both groups No adverse events were registered.	[182]
**OP-724** CREB-binding protein/β-catenin inhibitor	Phase 1	7 PBC patients median aged 68 years. Treatments: Group 1: OP-724 280 mg/m^2^/4 h/tw Group 2: OP-724 280 mg/m^2^/4 h/tw Only five of these completed twelve cycles of treatment. Consequently, the recommended dosage was determined to be 280 mg/m_2_/4 h.	**Primary** Assessment of the incidence of serious adverse events (SAEs). **Secondary** Measurement of the improvement in the modified Histological Activity Index (mHAI) score.	**Primary** SAEs did not occur. **Secondary** The most common AEs were abdominal discomfort (29%) and abdominal hepatic function (43%). Histological improvements in the fibrosis stage (2/5 40%) and mHAI score (3/5 60%).	[183]
**Setanaxib** NADP oxidase 1/4 inhibitor	Phase 2	111 patients with ≥6 months of UDCA treatment. Treatments: Group 1: oral setanaxib 400 mg/d (n = 38) Group 2 oral setanaxib 400 mg/b.d. (n = 36) Group 3: placebo (n = 37).	**Primary** Assessment of percentage change from baseline in GGT at Week 24.	**Primary** 104/111 patients completed Week 24. The primary end point was not met: change in GGT to Week 24 was −4.9% for Group 1 patients, −19.0% for Group 2 and −8.4% for placebo.	[184]
**Ursodeoxycholic acid (UDCA)**		73 PBC patients with poor response or who did not respond completely to a standard dose of UDCA. Treatments: Group 1: standard dosage of 13–15 mg/kg/d Group 2: higher dosage of 18–22 mg/kg/d.	**Primary** Evaluation of the rate of response at 6 months and drug side effects. **Secondary** Evaluation of the rate of response at 12 months and drug side effects.	**Primary** At 6 months, Group 2 patients achieved a response rate of 59.4% compared with 36.1% in the first group (*p* < 0.05) **Secondary** At 12 months, the Group 2 achieved a response rate of 59.4% compared with 47.2% in the Group 1 (*p* > 0.05).	[185]
**Budesonide/UDCA**	Phase 3	62 PBC patients after at least 6 months of UDCA terapy and hepatic inflammatory activity as assessed by Ishak score, and ALP >1.5 × ULN. Treatments: Group 1: budesonide 9 mg/d plus UDCA 12–16 mg/kg/d Group 2: placebo plus UDCA 12–16 mg/kg/d.	**Primary** Assessment of an improvement in liver histology with respect to inflammation and no progression of fibrosis. **Secondary** Measurement of changes in biochemical markers of liver injury.	**Primary** Comparing patients with paired biopsies only (n = 43), the primary histologic endpoint was not met (*p* > 0.05). **Secondary** Group 1 patients had a reduction of mean ALP and 35% of them achieved normalization of ALP (placebo 9%) (*p* < 0.05). Serious adverse events occurred in 10 patients receiving budesonide and 7 patients receiving placebo.	[186]
**A4250** IBAT inhibitor	Phase 2	9 patients with PBC, after a two-week whash out of bile acid sequestrant, treatment of cholestatic pruritus. Treatments: Group 1: A4250 0.75 mg (n = 4); Group 2: A2450 1.5 mg (n = 5).	After 4 weeks, evaluation of the effect of A4250 on pruritus, assessed by Visual Analogue Scale (VAS), 5D-itch scale and the pruritus module of the PBC40 questionnaire.	All 9 patients had an improvement in pruritus, until none or mild according to 5D-itch, VAS and PBC40 pruritus. Study was not completed due to abdominal pain (5/5) and diarrhoea (4/5).	[187]
**Bezafibrate** PPAR	Phase 2	74 cholestatic patients (24 PBC, 44 PSC, 2 SSC) with moderate to severe pruritus (≥5 of 10 on VAS). Treatments: Group 1: benzafibrate 400 mg/d Group 2: placebo.	**Primary** After 21 days, reduction of pruritus ≥ 50% in Group 1 patients. **Secondary** Evaluation of pruritus changes through VAS and 5D-Itch questionnaire. Evaluation of biochemical features changes.	70/74 patients completed the trial **Primary** Group 1 patients had a reduction of 45% (41% PSC, 55% PBC) and Group 2 of 11% to ≥50% reduction of severe or moderate pruritus (*p* < 0.05). **Secondary** Group 1 exhibited a reduction of morning (*p* < 0.05 vs. placebo) and evening (*p* < 0.05) VAS and improved the validated 5D-Itch questionnaire (*p* < 0.05 vs. placebo) compared with Group 2 patients.	[188]
**Rifampin/sertraline** PXR/SSRIs	Phase	36 patients with PSC and PBC. Treatments: Group 1: sertraline 100 mg/d (n = 18); Group 2: rifampin 300 mg/d (n = 18).	End points: pruritus severity, ALT, AST, ALP and total bilirubin at baseline and after 4 weeks of treatment.	No difference between sertraline and rifampin on pruritus improvement and total bilirubin.	[189]

## 8. Ongoing Clinical Trial PBC 2018–2023

In addition to the studies shown in Table 3, the consultations of the clinical trials website (https://clinicaltrials.gov/) provide a number of ongoing additional studies in patients with PBC. Some of these trials are listed in Table 4.

## 9. Conclusions

UDCA at a dose of 15 mg/kg (or higher) remains the cornerstone of treatment for PBC. In case of an incomplete response or intolerance, which is uncommon, a second-line therapy could be initiated with a choice between OCA, elafibranor and seladelpar [179]. An incomplete response is currently defined as an ALP level > 1.6× ULN or abnormal levels of bilirubin when a correct dose of UDCA (at least 15 mg/kg) is given for 12 months. It is expected that a combination of UDCA with novel second-line drugs or a combination of novel therapies with a more robust immune-mediated effect will make it possible to reach a complete normalization of markers of cholestasis including ALP. Whether this will indicate a disease cure remains to be determined.

In recent years OCA, a FXR agonist, first approved in 2016 as a second-line therapy for PBC, has shown efficacy in reducing ALP, but side effects associated with this agent seem to preclude its further use [190], and in June 2024, the EMA recommended revoking conditional marketing authorization for OCA in the EU (https://www.ema.europa.eu/en/news/ema-recommends-revoking-conditional-marketing-authorisation-ocaliva accessed on 29 August 2024). The EMA’s human medicines committee (CHMP) has recommended that the marketing authorization for OCA, “be revoked, because its benefits are no longer considered to outweigh its risks”. More specifically, at the time of its conditional marketing authorization in 2016, OCA was shown to reduce the ALP and bilirubin in patients with PBC, and this was considered indicative of an improvement in the condition of the liver. However, the clinical benefits of OCA needed to be demonstrated in further studies, which were requested by the EMA as part of the conditions for granting marketing authorization to the medicine. Study 747-302, a Phase 4, Double Blind, Randomized, Placebo Controlled, Multicenter Study Evaluating the Effect of OCA on Clinical Outcomes in Subjects with Primary Biliary Cholangitis (COBALT Study) was a randomized clinical trial aimed at confirming the clinical benefits and safety of OCA in PBC patients that were resistant or intolerant to UDCA. The conclusions of the panel were that: “after reviewing the available evidence, the committee concluded that the clinical benefits of OCA have not been confirmed”. In particular, study 747-302 failed to show that OCA was more effective than a placebo in terms of the number of patients whose disease worsened or who died, both in the overall population and in a group of patients with early stage PBC (https://www.ema.europa.eu/en/news/ema-recommends-revoking-conditional-marketing-authorisation-ocaliva accessed on 29 August 2024).

Other FXR agonists are currently under evaluation, including cilofexor and tropifexor, but these agents also seem to induce pruritus. In Europe, the pan-PPAR agonist bezafibrate is frequently used off label as a second-line therapy for PBC [191]. The recent approval of elafibranor and seladelpar, however, opens novel perspectives for a more individualized approach using a personalized combination of drugs based on whether the patient is in an early disease stage, whether there is fibrosis or if the patient suffers from pruritus or severe fatigue.

## Figures and Tables

**Table 1:** Assessment of clinical response to UDCA in various clinical trials.

Name	Response Criteria
GLOBAL-PBC [161]	Bilirubin, ALP, albumin and platelet count after 12 months of UDCA and age at baseline
Paris-I [162]	ALP > 3 × upper normal values or AST > 2 × upper normal values or bilirubin > 1.0 mg/dL after 12 months of UDCA
Paris-II [163]	All three of the following: ALP > 1.5 × upper normal values, AST 1.5 × upper normal values, bilirubin > 1 mg/dL after 12 months of UDCA
Rotterdam [164]	Bilirubin > 1 × upper normal values and/or albumin < 1 × upper normal values afer 12 months UDCA
Barcelona [165]	Decrease in ALP < 40% and ALP > 1.0 × upper normal values after 12 months of UDCA
Toronto [166]	ALP > 1.67 × upper normal values after 24 months UDCA
Rochester [167]	ALP > 2 × upper normal values after 6 months or 12 months of UDCA

**Table 2:** Current and investigational therapies in PBC.

Intervention/Target	Mechanism(s)	Drug
**Bile acids**	Various mechanisms, including increased bile acid flow and immunemodulation	UDCA and norUDCA
**Antifibrotic agents** **NOX1/4 inhibitor**	Prevents generation of ROS and transformation of hepatic stellate cells in myo-fibroblasts	Setanaxib
**Apical Sodium BA Transporter (ABST) Inhibitors**	Mitigates the reabsorption of conjugated BAs from the ileum	A3907 Volixibat Linerixibat
**FXR agonist**	Various mechanisms	Linafexor, Cilofexor OCA, TQA3526 ASC42
**PPAR agonists**	Various mechanisms including immune modulation	Elafibranor (α/δ) Seladelpar (δ) Benzafibrate (α) Fenofibrate (α) Saroglitazar (α/γ)
**Anti-pruritogens** ○ **MrgprX4 antagonist** ○ **Opioid receptor agonists**	Attenuates itching induced by pruritogens (e.g., BAs, bilirubin) Agosnitsfor the opioid K receptors	EP547 Difelikefalin (CR845)
**HMG-CoA reductase inhibitors**	Lowers cholesterol production and reduces dyslipidemia-associated complications	Atorvastatin Rosuvastatin Simvastatin
**Gut microbiome-based therapies**	Shifts the gut microbiota profile toward a lower abundance of pathological species while promoting taxa responsible for immune tolerance	Probiotics and Fecal microbiota transplantation
**IMPDH inhibitor**	Prodrug of mycophenolic acid (MPA), limits de-novo synthesis of guanosine nucleotides	Mycophenolate
**Calcineurin inhibitor**	Immunosuppressant activity	Cyclosporine A
**JAK1/2 inhibitor**	Reduces cell proliferation, shows immunosuppressant activity and induces cell apoptosis	Baricitinib (LY3009104)

**Table 4:** Registered clinical trials.

Treatment	Clinical Trial	Treatments	End Points
**Benzafibrate (BZF)** PPARα	NCT04514965 Phase N.A.	PBC patients with inadequate response to UDCA. Group 1: BZF, dosages not given	**Primary** Assessment of sCD163 macrophages marker and other fibrosis markers levels, liver stiffness and bile acid composition from 4 weeks up to 3 years **Secondary** assessment of itching degree from 4 weeks up to 3 years
**Benzafibrate (BZF)** PPARα	NCT04751188 Phase III	PBC patients with inadequate response to UDCA. Group 1: Benzafibrate 200 mg/b.d. + UDCA 13–15 mg/kg/d Group 2: Placebo Comparator: Placebo b.d. + UDCA 13–15 mg/kg/d	**Primary** Evaluation of biochemical response as the reduction of ALP ≤ 1.5-fold the upper limit of normal (ULN), AST ≤ 1.5-fold the ULN and Bilirubin ≤ 1 mg/dL at 6 months **Secondary** Assessment of quality of life and pruritus intensity using visual analogue scales after 6 months
**Fenofibrate** PPARα	NCT05749822 Phase II/III	PBC with compensated cirrhosis and inadequate biochemical response to UDCA. Group 1: Placebo Comparator: Placebo + UDCA 13–15 mg/kg/d Group 2: Fenofibrate 200 mg/d + UDCA 13–15 mg/kg/d	**Primary** Evaluation of serum ALP levels normalization at 48 weeks **Secondary** Evaluation of serum ALP levels normalization; changes in pruritus and fatigue; onset of biological or clinical AEs (increased creatinine, blood urea nitrogen, creatine kinase, AST, ALT) at 4, 12, 24, 36 and 48 weeks
**Fenofibrate** PPARα	NCT05751967 Phase III	PBC patients with inadequate biochemical response to UDCA. Group 1: Placebo Comparator: Placebo + UDCA 13–15 mg/kg/d Group 2: Fenofibrate 200 mg/d + UDCA 13–15 mg/kg/d	**Primary** Assessment of ALP and total bilirubin normalization at 48 weeks **Secondary** Assessment of ALP and total bilirubin normalization; changes in fatigue, pruritus and quality of life; drug-related adverse events onset; survival rates without liver transplantation or liver decompensation, pruritus, portal hypertension and others at 4, 12, 24, 36 and 48 weeks
**Fenofibrate** PPARα	NCT06174402 Phase II/III	PBC patients. Group 1: Fenofibrate 200 mg/d + UDCA 13–15 mg/kg/d Group 2: Placebo Comparator: Placebo + UDCA 13–15 mg/kg/d	**Primary** Assessment of ALP levels normalization at 48 weeks **Secondary** Assessment of ALP levels normalization; changes in pruritus and fatigue (VAS scale); biological or clinical adverse events onset (creatinine, AST and ALT increase); hepatic impairment development at 4, 12, 24, 36 and 48 weeks
**Obeticholic Acid (OCA)** **Benzafibrate (BZF)**	NCT05239468 Phase II	PBC patients. Group 1: Double Blind phase treatment A: BZF 100 mg/d + 1 OCA Placebo + 1 BZF Placebo Group 2: Double Blind phase treatment B: BZF 400 mg/d + 1 OCA Group 3: Double Blind phase treatment C: OCA 5 mg/d + BZF 100 mg/d + 1 BZF Placebo Group 4: Double Blind phase treatment D: OCA 5 mg/d + BZF 400 mg/d Group 5: Long Term Safety Extension (LTSE) Phase treatment D: OCA 5 mg/d + BZF 400 mg/d	**Primary** Evaluation of ALP levels change at 2, 4, 6, 8, 10 and 12 weeks **Secondary** Assessment of percentage changes in ALP levels; AST, ALT, GGT, total and conjugated bilirubin and lipid pool normalization; changes in bile acids plasma values at 2, 4, 6, 8, 10 and 12 weeks
**Obeticholic Acid (OCA)** **Benzafibrate (BZF)**	NCT04594694 Phase II	PBC patients. Group 1: Treatment A: BZF 200 mg/d Immediate Release (IR) + 1 OCA Placebo + 1 BZF 400 mg/d Placebo Group 2: Treatment B: BZF 400 mg/d SR + 1 BZF 200 mg/d Placebo + 1 OCA Placebo Group 3: Treatment C: OCA 5 to 10 mg/d + BZF 200 mg/d IR + BZF 400 mg/d Placebo Group 4: Treatment D: OCA 5 mg to 10 mg + BZF 400 mg/d SR + BZF 200 mg/d Placebo Group 5: LTSE phase, OCA + BZF: participants will continue the original treatment assigned but OCA and BZF dose may be optimized based on safety and efficacy	**Primary** Evaluation of ALP levels at day 1 and 4, 8 and 12 weeks **Secondary** Percentage assessment of ALP, AST, ALT, GGT normalization at day 1 and 4, 8 and 12 weeks; change in total and conjugated bilirubin, lipid and bile acids pool at day 1 and 4, 8 and 12 weeks
**Obeticholic Acid (OCA)**	NCT05450887 Phase III	PBC patients. Group 1: OCA 5 to 10 mg/d + UDCA 13–15 mg/kg/d if already receiving UDCA; if the subjects could not tolerate UDCA, they were not treated with UDCA Group 2: Placebo Comparator + UDCA 13–15 mg/kg/d if already receiving UDCA; if the subjects could not tolerate UDCA, they were not treated with UDCA	**Primary** Evaluation of ALP ≤ 1.67-fold the ULN, ALP decrease ≥ 15% from baseline and total bilirubin ≤ ULN up to 12 months **Secondary** Assessment of absolute and percentage change of ALP, AST, ALT, GGT, total and direct bilirubin; quality of life evaluation via PBC-40 score percentage change at 3, 6, 9 and 12 months
**Volixibat** ASBT inhibitor	NCT05050136 Phase II	Group 1: Volixibat 20 mg/b.d. Group 2: Volixibat 80 mg/b.d. Group 3: Placebo	**Primary** Assessment of mean change in the daily itch scores using the Adult Itch Reported Outcome (Adult ItchRO) questionnaire up to week 28 **Secondary** Evaluation of ALP, total bilirubin, serum bile acids levels change; adverse events incidents; assessment of quality of life (PBC-40 score), fatigue and sleep disturbance (PROMIS^®^) up to 28 weeks
**Linerixibat** IBAT imnhibitor	NCT04950127 Phase III	PBC patients. Group 1: Linerixibat, dosages not given Group 2: Linerixibat followed by Placebo, dosages not given Group 3: Placebo Group 4: Placebo followed by Linerixibat, dosages not given	**Primary** Assessment of change from baseline in Monthly Itch Scores using Numerical Rating Scale (NRS) over 24 weeks **Secondary** Evaluation of changes in Mean Worst Daily Itch score at Week 2. Changes in PBC-40 score, PGI-S, PGI-C and Monthly Sleep Score, measured by NRS; reduction in the Monthly Itch Score; changes in ALP and bilirubin levels up to 24 weeks
**Linerixibat** Ileal Bile Acid Transporter Inhibitor (IBAT)	NCT04167358 Phase III	Patients with PBC Group 1: Linerixibat in participant who previously participated in the Phase 2 studies (BAT117213 and 201000 GLIMMER [Group 1]) and Phase 3 study (212620 GLISTEN [Group 2]), dosages not given	**Primary** Assessment of AEs and SAEs onset up to 66 months **Secondary** Changes in PBC-40 score, in health-related quality of life (EQ-5D-3L score) and self-related health (EQ VAS score); assessment of depression intensity (BDI-II score); changes in hematology, biochemistry and coagulation parameters up to 65 months. Changes in pruritus (MIS-NRS), fatigue (MFS-NRS) and sleep (MSS-NRS) up to week 52 of continuous treatment
**Obeticholic Acid (OCA)** **UDCA**	NCT04956328 Phase III	PBC patients with inadequate response to UDCA. Group 1: OCA 5–10 mg/d + UDCA (continue pre-study dose) for 24 weeks and then titrating up to 10 mg based on tolerability and response Group 2: Placebo + UDCA (continue pre-study dose) for 48 weeks	**Primary** Percentage of patients with ALP < 1.67-fold the ULN, ALP decreased at least 15% and total bilirubin ≤ ULN up to 48 weeks **Secondary** Percentage of patients with ALP < 1.67-fold the ULN, ALP decreased at least 15% and total bilirubin ≤ ULN at 4, 12, 24 and 36 weeks. Assessment of rate of change of AST, ALT, ALP, GGT, total bile acids and total bilirubin ad liver function indicators up to 48 weeks
**UDCA** **Total Glucosides of Peony (TGP)** Anti-inflammatory and immune regulatory effects	NCT04618575 Phase IV	PBC patients with Autoimmune Hepatitis (AIH) 1. Group 1: UDCA + TGP, dosages not given Group 2: UDCA only, dosages not given	**Primary** Percentage of patients in biochemical remission defined as normalization of serum ALT and IgG levels after 24 weeks and up to 12 months **Secondary** Assessment of patients in partial remission (AST/ALT > 1-fold the ULN and <2-fold the ULN), with minimal response (AST/ALT still > 2-fold the ULN) or with treatment failure; drug-related side-effects and clinical symptoms (jaundice, fatigue, itching) onset; changes in the proportion of blood immune cells (% of T cells, DCs, Treg, NK.) up to 12 months
**UDCA** **Low-Dose Glucocorticoid (GC)** Decrease in symptoms severity	NCT04617561 Phase IV	PBC patients with Autoimmune Hepatitis (AIH) 2. Group 1: UDCA 13–15 mg/kg/d Group 2: UDCA 13–15 mg/kg/d + Methylprednisolone 12 mg/d in induction phase (2–4 mg/d in maintenance phase)	**Primary** Percentage of patients in biochemical remission defined as normalization of serum ALT and IgG levels up to 12 months **Secondary** Assessment of patients in partial remission (AST/ALT > 1-fold the ULN and <2-fold the ULN), with minimal response (AST/ALT still > 2-fold the ULN) or with treatment failure up to 12 months. Drug-related side-effects onset and changes in the proportion of blood immune cells (% of T cells, DCs, Treg, NK.) at 12 months. Assessment of AST, ALT and IgG serum levels at 3, 6 and 12 months
**Saroglitazar Magnesium** PPARα/γ	NCT05133336 Phase III	PBC patients. Group 1: Saroglitazar Magnesium 2 mg/d Group 2: Saroglitazar Magnesium 1 mg/d Group 3: Placebo	**Primary** Assessment of number of subjects with biochemical response as ALP < 1.67-fold the ULN, ALP decrease ≥ 15% from baseline and total bilirubin ≤ ULN (or direct bilirubin ≤ ULN in patients with known Gilbert’s Syndrome) up to 52 weeks **Secondary** Assessment of number of subjects with biochemical response as ALP < 1.67-fold the ULN, ALP decrease ≥ 15% from baseline and total bilirubin ≤ ULN (or direct bilirubin ≤ ULN in patients with known Gilbert’s Syndrome) at 4, 8, 16 and 24 weeks. Percentage improvement or normalization in ALP values; improvement in liver stiffness measurement of at least 25% via FibroScan^®^; changes in liver enzyme (AST, ALT, GGT, total bilirubin and albumin) and lipid (TG, LDL-C, HDL-C, total cholesterol) parameters; changes in serum bile acids at 24 and 52 weeks. Assessment of changes in health-related quality of life (PBC-40 score) and itching (5D scale, PGI-C scale, PGT-B scale, PGI-Worst Itch Severity scale) at 4, 8, 16, 24 and 52 weeks. Assessment of treatment-related AEs, SAEs, AEs of special interest (e.g., DILI) onset; significant changes in clinical laboratory test results (hematology, biochemistry, urinalysis), in vital signs, in ECG and in body weight at 52 weeks
**Setanaxib** NADP oxidase (NOX) 1/4 inhibitor	NCT05014672 Phase III	PBC patients. Group 1: Setanaxib 1200 mg/day. Eventual escalation to 1600 mg/day will be determined for the extension period Group 2: Setanaxib 1600 mg/d. Eventual reduction to 1200 mg/day mg/day will be determined for the extension period Group 3: Placebo. During the extension period, participants will switch from placebo to Setanaxib at a dose of either 1200 or 1600 mg/d depending on interim analysis outcome	**Primary** Assessment of biochemical response as ALP < 1.67-fold the ULN, ALP decrease ≥ 15% from baseline and total bilirubin ≤ ULN up to 52 weeks **Secondary** Assessment of changes in fatigue (PROMIS^®^, PBC-40 score, PGI-S, PGI-C), liver stiffness (FibroScan^®^), itching (WI-NRS, PBC-40, PGI-S, PGI-C); TEAEs and AESIs onset up to 52 weeks
**HTD1801 (BUDCA)** Hypolipidemic agent	NCT04604652 Phase II	PBC patients with inadequate response to standard UDCA therapy. Group 1: HTD1801 (BUDCA) 2000 mg/d	**Primary** Evaluation of changes in serum ALP at 12 weeks **Secondary** Assessment of serum bilirubin, GGT, total cholesterol, LDL-C, tryglicerides and inflammatory markers (fibrinogen, CRP, haptoglobin, IgG) changes; itching variations (Pruritus VAS), AEs onset as well as changes in physical examinations, vital signs and clinical laboratory values at 12 weeks
**TQA3526** FXR	NCT04278820 Phase II	PBC patients. Group 1: Climbing Group: TQ3526 drug or Placebo once daily, dosages not given Group 2: Titration Group: TQ3526 drug or Placebo once daily, dosages not given Group 3: Extension Group: TQ3526 drug or Placebo once daily, dosages not given	**Primary** Evaluation of ALP levels reduction up to 24 weeks **Secondary** Assessment of ALP, ALT, AST, GGT, total bilirubin, LDL-C, HDL-C, TG and TC at 2, 4, 8, 12, 14, 16, 20 and 24 weeks. Assessment of Cmax and Tmax. Evaluation of TEAEs and SAEs onset up to 24 weeks
**ASC42** FXR	NCT05190523 Phase II	PBC patients. Group 1: ASC42 5 mg/d Group 2: ASC42 10 mg/d Group 3: ASC42 15 mg/d Group 4: Placebo	**Primary** Evaluation of percentage changes in ALP levels at day 85 **Secondary** Evaluation of percentage and absolute changes of ALP, GGT, ALT, AST; incidence of TEAEs, SAEs and AESI onset at day 15, 29, 57 and 85
**EP547** MAS related GPR family member X4 (MrgprX4)	NCT05525520 Phase II	PBC or PSC patients with cholestatic pruritus. Group 1: EP547 100 mg/d Group 2: Placebo	**Primary** Evaluation of changes in pruritus (WI-NRS) up to 6 weeks **Secondary** Evaluation of changes and reduction in pruritus (5D-Itch scale, PGI-C, PGI-S); assessment of AEs onset; measurement of Cmax up to 6 weeks
**Probiotics (Micro V Probiotics)**	NCT03521297 Phase II	PBC patients with inadequate response to UDCA. Group 1: Placebo + SOC UDCA 13–15 mg/kg/d Group 2: Oral administration three times per day of Probiotics + SOC UDCA 13–15 mg/kg/d	Assessment of percentage of patients with biochemical response as serum ALP or GGT decreased by 20% from baseline after 6 months
**Mycophenolate Mofetil** IMPDH inhibitor **Cyclosporin A** Calcineurin inhibitor/immunosuppressive agent	NCT04376528 Phase IV	PBC patients with PBC-AIH overlap syndrome and nonresponsive to UDCA standard therapy. Group 1: Cyclosporin A + UDCA SOC, dosages not given Group 2: Mycophenolate Mofetil + UDCA SOC, dosages not given	**Primary** Evaluation of percentage of patients in biochemical remission as normalization of serum ALT and IgG levels after 24 weeks and up to 6 months **Secondary** Evaluation of partial remission (AST or ALT serum levels > ULN and <2-fold ULN), minimal response (AST or ALT still > 2-fold ULN) or treatment failure; assessment of changes in liver stiffness (shear-wave elastography); drug-related side effects onset up to 6 months
**CNP-104** Immunomodulating agent	NCT05104853 Phase I/II	PBC patients non-responsive to UDCA and/or OCA. Group 1: 200 mL intravenous infusion of CNP-104 4 mg/kg on day 1 and day 8 Group 2: 200 mL intravenous infusion of CNP-104 8 mg/kg on day 1 and day 8 Group 3: Placebo Comparator	**Primary** Assessment of AEs and SAEs onset; laboratory tests (hematology, serum chemistry, coagulation panel, urinalysis) through study completion, an average of 720 days. Assessment of serum cytokines (TNFα, IL-4, IL-6, IL-10, IL-1β, MCP-1, IFN-γ) for an average of 15 days. Evaluation of ALP changes at day 60 **Secondary** Evaluation of changes in AMA and liver fibrosis (FibroScan^®^) at day 90 and 720. Changes in modified PBC-40 score, Weekly Mean Itch Score, liver enzyme levels (albumin, bilirubin, AST, ALT, GGT) and antigen-specific CD4^+^/CD8^+^ T cells asset at day 60 and 720. Assessment of ALP levels at day 720.
